# QuickStats

**Published:** 2015-06-12

**Authors:** FROM THE NATIONAL CENTER FOR HEALTH STATISTICS

**Figure:**
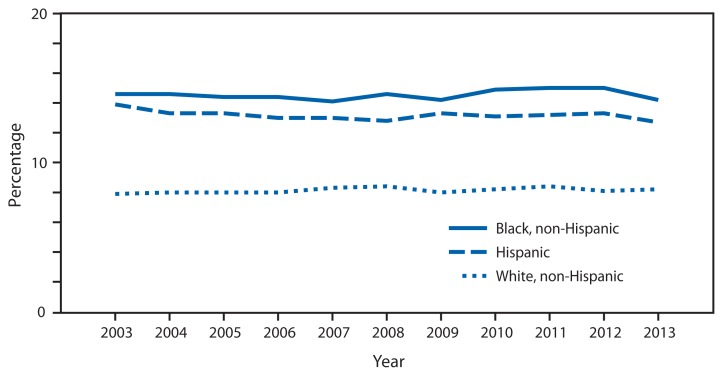
Age-Adjusted^*^ Percentage of Persons Who Reported Fair or Poor Health,^†^ by Race and Hispanic Origin — National Health Interview Survey, United States,^§^ 2003–2013 ^*^ Estimates are age-adjusted to the 2000 projected U.S. standard population using six age groups (in years): <18, 18–44, 45–54, 55–64, 65–74, ≥75. ^†^Respondents were asked “Would you say (person’s) health in general is excellent, very good, good, fair, or poor?” Responses of fair or poor were combined into one measure. ^§^Estimates are based on household interviews of a sample of the noninstitutionalized U.S. civilian population.

During 2003–2013, non-Hispanic black and Hispanic persons were more likely than non-Hispanic white persons to report fair or poor health. Fair or poor health status ranged between 14%–15% for non-Hispanic black persons and 13%–14% for Hispanic persons, and was 8% for non-Hispanic white persons, with no significant changes during the decade in the percentage of those reporting fair or poor health within each of the three groups.

**Source:** National Center for Health Statistics, *Health, United States, 2014, With Special Feature: Adults Aged 55–64* (Table 50). Available at http://www.cdc.gov/nchs/hus.htm.

**Reported by:** Mary Ann Bush, MS, mbush@cdc.gov, 301-458-4130, and Shilpa Bengeri.

